# ASO Author Reflections: Rethinking Clinical Nodal Staging in Breast Cancer

**DOI:** 10.1245/s10434-026-20179-1

**Published:** 2026-07-20

**Authors:** Pooja M. Varman

**Affiliations:** Cleveland Clinic Foundation, Cleveland, OH USA

## Past

Clinical nodal staging in breast cancer has long relied on physical examination findings of mobile or fixed axillary lymph nodes rather than the number of suspicious nodes identified on pretreatment imaging.^1^ The current AJCC staging system classifies all patients with mobile, biopsy-confirmed axillary nodal metastases as cN1, regardless of whether one or six nodes appear suspicious on ultrasound or MRI.^2^ This stands in contrast to pathologic staging, which stratifies patients by node count with well-established prognostic implications. Modern breast imaging routinely documents the number and morphology of suspicious axillary nodes; yet this information does not factor into clinical staging and is not required by current ACR reporting guidelines. Whether imaging-defined nodal burden within the cN1 category carries meaningful prognostic information was the central question motivating this work.

## Present

In this single-institution retrospective cohort of 618 patients with node-positive breast cancer treated with neoadjuvant chemotherapy (NAC), we subcategorized cN1 patients by pretreatment imaging node count into cN1a (1–3 suspicious nodes) and cN1b (≥ 4 suspicious nodes) and compared their pathologic outcomes against a cN2a reference group. We found that cN1b patients were nearly twice as likely to be upstaged to ypN2 or higher after NAC compared with cN1a patients (24.1% vs. 12.8%; odds ratio 1.958, *p* = .009), and their average residual nodal burden (4.6 positive nodes) aligned far more closely with cN2a disease (5.5 nodes) than with cN1a (2.8 nodes). Pairwise analyses showed that cN1b and cN2a were statistically indistinguishable from each other on key pathologic endpoints, while both differed significantly from cN1a. Despite these pathologic differences, 3-year DFS and OS did not differ significantly across groups at a median follow-up of 41.4 months, and nodal group was not an independent predictor of survival on multivariable analysis. This study contributes to a growing body of evidence that pretreatment imaging provides clinically meaningful prognostic information beyond current AJCC cN staging.

## Future

Prospective validation of imaging-based cN1 subcategorization in larger, multi-institutional datasets is needed, with longer follow-up to determine whether the pathologic differences between cN1a and cN1b translate into meaningful differences in locoregional recurrence or survival. Future work should examine whether cN1b patients derive differential benefit from more aggressive axillary staging strategies, such as targeted axillary dissection, or from escalated regional radiation. Integration of imaging node count into standardized radiology reporting, as proposed by emerging frameworks, such as Node-RADS,^3^ would be a practical first step toward making this information consistently available for clinical and research use.^4,5^ Ultimately, formal evaluation of whether the AJCC cN1 category should be subcategorized by imaging-defined nodal burden is warranted and could refine risk stratification, surgical planning, and trial eligibility criteria for a meaningful subset of patients with node-positive breast cancer.

## References

[1] Varman PM, Taft DA, Patel M, et al. Is it time to redefine clinical nodal staging for breast cancer? *Ann Surg Oncol*. 2026. 10.1245/s10434-026-20128-y. PMC13630902 42461556

[2] Amin MB, Greene FL, Edge SB, et al. AJCC cancer staging manual: continuing to build a bridge from a population-based to a more “personalized” approach to cancer staging. *CA Cancer J Clin*. 2017;67(2):93–9. 10.3322/caac.21388 28094848

[3] Elsholtz FHJ, Asbach P, Haas M, et al. Introducing the node reporting and data system 1.0 (Node-RADS). *Eur Radiol*. 2021;31(8):6116–24. 10.1007/s00330-020-07572-4 PMC8270876 33585994

[4] de Wild SR, de Munck L, Simons JM, et al. De-escalation of radiotherapy after primary chemotherapy in cT1-2N1 breast cancer (RAPCHEM). *Lancet Oncol*. 2022;23(9):1201–10. 10.1016/S1470-2045(22)00482-X 35952707

[5] Sun SX, Moseley TW, Kuerer HM, Yang WT. Imaging-based approach to axillary lymph node staging and sentinel lymph node biopsy in patients with breast cancer. *Am J Roentgenol*. 2020;214(2):249–58. 10.2214/AJR.19.22022 31714846

